# 26. NOVEL APPROACHES TO PSYCHOSIS RISK: MOVEMENT, STRESS MODULATION, REWARD AND LANGUAGE

**DOI:** 10.1093/schbul/sby014.105

**Published:** 2018-04-01

**Authors:** Cheryl Corcoran

**Affiliations:** Icahn School of Medicine at Mount Sinai

## Abstract

**Overall Abstract:** Research on psychosis risk now encompasses novel and innovative approaches for understanding not only positive symptoms, but also impairment in sensorimotor function, stress regulation, reward learning, and language. These include the use of machine learning and cluster analysis with resting state functional connectivity analyses, in vivo measures of dopamine function in response to stress, computational modeling, and automated natural language processing analyses in collaboration with IBM.

First, Vijay Mittal will describe subtypes of clinical risk, identifying a group with aggregated measures of sensorimotor dysfunction, developmental markers, negative symptoms and cognitive deficits, who have a discrete pattern of corticostriatal connectivity.

Second, Romina Mizrahi will present her results from a study of dopamine response to stress in prefrontal cortex, using positron emission tomography, and correlations with cortisol release, across stages of illness, including schizophrenia and clinical risk, with healthy volunteers for comparison.

Third, James Waltz will present data on the computational processes that may underlie both positive and negative symptoms, in respect to dopamine-based signals of salience. These include aberrant or erratic salience signaling, as well as a decreased ability to identify relevant salient stimuli, which could impair reward learning and motivation. His cohort includes individuals with psychosis, and those at clinical risk for it, as well as non-psychosis patient controls.

Fourth, Cheryl Corcoran will describe the use of automated natural language processing (NLP), with machine learning (ML) to identify semantic and syntactic features that predict psychosis onset. She will show data on cross-validation of the classifier in a second risk cohort, and its correlation with demographics and manual linguistic features. Overall, there is an apparent norm of semantic coherence and syntactic complexity from which individuals with psychosis deviate, even prior to its onset.

Finally, the discussant will review these data in the context of his experience and ongoing leadership in the field of psychosis risk research, leading audience discussion, and outlining a roadmap for future research in the field.

